# TRIM13 Positively Regulates the NF-κB Signaling Pathway Induced by Encephalomyocarditis Virus

**DOI:** 10.3390/v18040466

**Published:** 2026-04-14

**Authors:** Xiaolan Ji, Donglin Bi, Mingqi Liu, Xiangru Du, Zhiqi Wang, Haiqing Li, Jinluan Wang, Yiyang Fan, Hao Gao, Derong Zhang, Jialin Bai, Qiongyi Li

**Affiliations:** 1 Key Laboratory of Biotechnology and Bioengineering of State Ethnic Affairs Commission, Biomedical Research Center, Northwest Minzu University, Lanzhou 730030, China; jxl18226033901@163.com (X.J.); bdlwym@163.com (D.B.); 13595842748@163.com (M.L.); 13147837395@163.com (X.D.); wang12080682@163.com (J.W.); potato7@outlook.com (H.G.); zhangdr@xbmu.edu.cn (D.Z.); 2 College of Life Science and Engineering, Northwest Minzu University, Lanzhou 730030, China; 13853418307@163.com (Z.W.); y242530472@stu.xbmu.edu.cn (H.L.); 13354925037@163.com (Y.F.); jlbai@xbmu.edu.cn (J.B.)

**Keywords:** tripartite motif 13, nuclear factor-κB, IκBα, encephalomyocarditis virus

## Abstract

Encephalomyocarditis virus (EMCV) belongs to the genus Cardiovirus of the family Picornaviridae. It is a non-enveloped, positive-sense, single-stranded RNA virus and an important pathogen causing encephalomyocarditis (EMC). Tripartite motif 13 (TRIM13) is a member of the tripartite motif (TRIM) family and serves as an important effector molecule in antiviral innate immunity. However, its antiviral activity and underlying molecular mechanisms during EMCV infection remain unknown. In this study, we identified TRIM13 as a regulator of NF-κB activation. TRIM13, dependent on its E3 ubiquitin ligase activity, directly binds to IκBα and dose-dependently increases its phosphorylation level. To determine the chain type of IκBα polyubiquitination, antibodies specific for K48-linked and K63-linked ubiquitin were used. Our data indicated that IκBα was subjected to polyubiquitination independent of K48 and K63 linkages. This interaction promotes non-K48/K63-linked polyubiquitination of IκBα, thereby inducing NF-κB nuclear translocation. Subsequently, nuclear NF-κB activates the secretion of pro-inflammatory cytokines, exacerbating inflammatory responses and ultimately facilitating EMCV infection.

## 1. Introduction

Encephalomyocarditis virus (EMCV), a member of the genus *Cardiovirus* within the family *Picornaviridae* [1], is a non-enveloped RNA virus that primarily infects mammals, including rodents and primates, via the faecal–oral route, leading to encephalomyocarditis (EMC) [2,3]. EMCV infection causes significant economic losses in the global swine industry [3]. Inflammation constitutes the immediate innate immune response to either pathogen invasion or sterile tissue injury; it is initiated upon recognition of pathogen-associated molecular patterns (PAMPs) derived from microbes and damage-associated molecular patterns (DAMPs) released by injured or necrotic host cells [4,5,6]. The nuclear factor-κB (NF-κB) signaling pathway serves as a central regulator of inflammatory responses, orchestrating the transcriptional activation of numerous pro-inflammatory genes [7]. EMCV infection triggers robust NF-κB-mediated inflammatory responses that contribute to pathogenesis [8]. While the activation mechanisms are well characterized, the negative regulatory mechanisms remain poorly understood. Tripartite motif-containing protein 13 (TRIM13) belongs to the tripartite motif (TRIM) protein family, also known as the RBCC family, characterized by the presence of a RING domain, one or two B-box domains, and a coiled-coil domain at the N-terminus, followed by a highly variable C-terminal domain. Based on differences in the C-terminal domain, the TRIM family is classified into eleven subgroups [9]; TRIM13 contains only one B-box 2 domain, and its C-terminus comprises a transmembrane (TM) region that anchors the protein to the endoplasmic reticulum. The RING domain confers E3 ubiquitin ligase activity, enabling TRIM proteins to catalyze ubiquitination of target substrates. This RING domain-mediated ubiquitin ligase activity is central to the antiviral function of most TRIM proteins. TRIM13 is involved not only in innate antiviral immunity and autophagy but also plays a role in the pathogenesis of various autoimmune diseases [10,11,12] Although TRIM13 has been characterized as a tumor suppressor in non-small cell lung cancer (NSCLC) through inhibition of NF-κB signaling [13], its antiviral activity and underlying molecular mechanisms during EMCV infection remain largely unknown.

Based on these observations, we hypothesized that TRIM13 suppresses EMCV-induced NF-κB activation, thereby attenuating viral proliferation. Contrary to this prediction, our results revealed that TRIM13 paradoxically promotes EMCV replication by activating NF-κB signaling and upregulating IκBα expression. This study elucidates a previously unrecognized proviral function of TRIM13 and identifies a potential therapeutic target for EMCV-associated diseases.

## 2. Materials and Methods

### 2.1. Cells and Viruses

HeLa and BHK-21 cells were obtained from the Animal Cell Engineering Technology Research Center of Gansu Province (Lanzhou, China) and maintained in Dulbecco’s Modified Eagle Medium (DMEM; Lanzhou Minhai Biotechnology Engineering Co., Ltd., Lanzhou, China) supplemented with 10% (*v*/*v*) newborn bovine serum (NBS; Lanzhou Minhai Bio-Engineering Co., Ltd., Lanzhou, China). HEK293T cells were obtained from the same source and cultured in DMEM supplemented with 10% (*v*/*v*) fetal bovine serum (FBS; Lanzhou Minhai Bio-Engineering Co., Ltd.). All cells were incubated at 37 °C with 5% CO_2_. *Escherichia coli* DH5α competent cells were purchased from TransGen Biotech (Beijing, China). Encephalomyocarditis virus (EMCV; GenBank accession number X74312) and vesicular stomatitis virus (VSV) were obtained from the Key Laboratory of Bioengineering and Technology of National Ethnic Affairs Commission, Biomedical Research Center (Lanzhou, China).

### 2.2. Antibodies and Reagents

The following primary antibodies were used: mouse monoclonal anti-GAPDH antibody [6C5] (ab8245), rabbit monoclonal anti-TRIM13 antibody (ab138890), rabbit polyclonal anti-TRIM13 antibody (N-terminal) (ab72129), anti-ubiquitin antibody (13-1600), anti-K48-linked ubiquitin antibody (ab140601), anti-K63-linked ubiquitin antibody (ab179434), rabbit monoclonal anti-IκBα antibody [E130] (ab32518), rabbit monoclonal anti-phospho-IκBα (Ser36) antibody [EPR6235(2)] (ab133462), and mouse anti-EMCV VP1 monoclonal antibody (purchased from Key Laboratory of Bioengineering and Technology of National Ethnic Affairs Commission, Lanzhou, China). Additional primary antibodies included TBK1/NAK (E8I3G) rabbit mAb (#38066), phospho-TBK1/NAK (Ser172) (D52C2) XP^®^ rabbit mAb (#5483), IRF-7 antibody (#4920), phospho-IRF-7 (Ser471/472) antibody (#5184), MAVS (D5A9E) rabbit mAb (#14368), NF-κB p65 (D14E12) XP^®^ rabbit mAb (#8242), MDA-5 (D74E4) rabbit mAb (#5321), and phospho-NF-κB p65 (Ser536) (93H1) rabbit mAb (#3033). Horseradish peroxidase (HRP)-conjugated goat anti-rabbit IgG (#111-035-003) and goat anti-mouse IgG (#115-035-003) secondary antibodies were obtained from Jackson ImmunoResearch Laboratories (West Grove, PA, USA). Anti-GFP mouse monoclonal antibody (HT801) was purchased from TransGen Biotech (Beijing, China).

Cell culture reagents included 0.25% trypsin-EDTA solution (Lanzhou Bailing Biotechnology Engineering Co., Ltd., Lanzhou, China) and Opti-MEM^®^ Reduced Serum Medium (Thermo Fisher Scientific, Waltham, MA, USA). Transfection was performed using Lipofectamine™ 2000 Reagent (Thermo Fisher Scientific). Total RNA was extracted using the Cell Total RNA Extraction Kit (Accurate Biology, Changsha, China), and reverse transcription was performed with the Evo M-MLV Reverse Transcription Kit (including gDNA removal reagent for qPCR) (Accurate Biology).

For protein analysis, the BCA Protein Assay Kit (Thermo Fisher Scientific) and HyperSignal high-sensitivity ECL chemiluminescent substrate (Shanghai Yamei Biomedical Technology Co., Ltd., Shanghai, China) were used. Quantitative PCR was performed using SYBR™ Green Master Mix (Beijing Sizhengbai Biotechnology Co., Ltd., Beijing, China). Cytokine levels were measured using human IL-6, human TNF-α, mouse IL-6, and mouse TNF-α ELISA kits (mlbio, Shanghai, China). Luciferase assays were conducted using the Luciferase Assay System (Promega, Madison, WI, USA).

### 2.3. Plasmid and siRNA Transfections

The pcDNA3.1 empty vector and pcDNA3.1-TRIM13 expression plasmid were obtained from the Key Laboratory of Bioengineering and Technology of National Ethnic Affairs Commission Biomedical Research Center. Small interfering RNAs (siRNAs) targeting the gene of interest were purchased from Guangzhou Ruibo Biological Co., Ltd. (Guangzhou, China). Reporter gene plasmid (pGL3-NF-κB-luc and pGL3-IL-6-luc; expression plasmids pRK-Flag-MDA5, pRK-Flag- MAVS, pRK-Flag- TBK1, pRK-Flag- IRF7, pRK-Flag-TRAF3, pRK-Flag-TRAF6, pRK-Flag-IKKα, and pRK-Flag-IKKβ (Promega Corporation, Promega, USA)) HeLa cells were seeded into 6-well plates and cultured until reaching 70–80% confluence. Plasmids or siRNAs were then transfected into the cells using Lipofectamine™ 2000 (Thermo Fisher Scientific, Waltham, MA, USA) according to the manufacturer’s instructions. Cells were harvested 48 h post-transfection, and the efficiency of protein overexpression or knockdown was evaluated by Western blot analysis. The siRNAs used in this experiment are shown in Table 1.

### 2.4. Western Blot and Densitometric Analysis

Cells were lysed in NP-40 lysis buffer (1% NP-40, 150 mM NaCl, 50 mM Tris-HCl, pH 7.4) supplemented with 1 mM phenylmethylsulfonyl fluoride (PMSF) and heated at 95 °C for 10 min. Equal amounts of protein lysates were separated by 10% sodium dodecyl sulfate–polyacrylamide gel electrophoresis (SDS-PAGE) and subsequently transferred onto polyvinylidene difluoride (PVDF) membranes. After blocking with 5% (*w*/*v*) non-fat dry milk in Tris-buffered saline containing 0.1% Tween-20 (TBST) for 1 h at room temperature, the membranes were incubated overnight at 4 °C with specific primary antibodies. Following three washes with TBST, the membranes were incubated with horseradish peroxidase (HRP)-conjugated secondary antibodies (goat anti-rabbit or goat anti-mouse IgG, 1:5000 dilution) for 1 h at room temperature. Immunoreactive bands were visualized using an enhanced chemiluminescence (ECL) detection system and captured with a chemiluminescence imaging system (Cytiva, Shanghai, China). Band intensities were quantified by densitometric analysis using ImageJ software (version 1.54d, National Institutes of Health, Bethesda, MD, USA) and normalized to the corresponding internal control (e.g., β-actin or GAPDH) to ensure equal protein loading.

### 2.5. RT-qPCR

Total RNA was extracted using TAKARA ‘s TRIzol Reagent Kit (total RNA extraction kit) and reverse-transcribed using the Evo M-MLV Reverse Transcription Kit (including gDNA removal reagent for qPCR). RT-qPCR was performed using SYBRTM Green Master Mix. The relative mRNA expression was normalized to GAPDH. The fold modulation in gene expression was analyzed by 2^−ΔΔCt^ method. The primers of target genes are shown in Table 2.

### 2.6. Enzyme-Linked Immunosorbent Assay (ELISA)

The supernatant was harvested after cells had been stimulated with inflammatory cytokines. Interleukin-6 (IL-6) and Tumor Necrosis Factor-alpha (TNF-α) protein levels were estimated with an ELISA kit using rat anti-human IL-6 antibodies and TNF-α antibodies (mlbio, Shanghai, China), according to the manufacturer’s instructions.

### 2.7. Luciferase Assays

HeLa cells were placed in 24-well plates. When the cell density reached 70%, each well was transfected with an internal reference plasmid (pRL-TK (Promega Corporation, Promega, Madison, WI, USA)) and a reporter gene plasmid (pGL3-NF-κB-luc and pGL3-IL-6-luc; expression plasmids pRK-Flag-MDA5, pRK-Flag-MAVS, pRK-Flag-TBK1, pRK-Flag-IRF7, pRK-Flag-TRAF3, pRK-Flag-TRAF6, pRK-Flag-IKKα, and pRK-Flag-IKKβ (Promega Corporation, Promega, Madison, WI, USA)) in turn. The cells were cultured for 24 h, and the experiment was carried out according to the operating instructions of the Promega luciferase detection kit. After washing the sample with PBS, it needed to be fully mixed with 100 μL of cell lysate, the culture plate was gently shaken at room temperature for 15 min, and the lysate was transferred to the new EP tube. A 100 μL volume of LLARII was added to the 96-well plate in the dark, and then 20 μL of lysate was added. Each test group was set up with three replicate holes and placed in the luciferase detector, the activity of firefly luciferase was detected, and the data were recorded. Subsequently, 100 μL Stop & Glo^®^ Reagen (Promega, Madison, WI, USA) was added to the hole and placed in a luciferase detector to detect the activity of renilla luciferase and record the data.

### 2.8. Immunofluorescence Assay

The infected cells were treated with 75% ethanol for 1 h at 4 °C. Following a PBS wash, the cells were exposed to a primary antibody at 37 °C for 1 h. Afterward, the cells were washed again and incubated with a secondary antibody for 1 h at 37 °C. Finally, the cells were fixed using mounting medium containing DAPI and examined under a Carl Zeiss LSM 880 confocal microscope (Carl Zeiss, Oberkohen, Germany).

### 2.9. Virus Replication

HeLa cells were incubated with EMCV at a multiplicity of infection (MOI) of 1 for 1 h at 37 °C to allow for virus attachment to the cell surface. The cells were then washed multiple times with PBS to remove any unabsorbed EMCV particles. After 9 h of culture at 37 °C in EMCV containing 2.5% NBS, culture supernatant collected at 24 h was used for TCID50 detection.

### 2.10. TCID50 Analysis

HeLa cells were seeded in 96-well plates 24 h prior to the experiment. Following reaching full confluence, the virus was serially diluted by a factor of 10 and then seeded into 96-well plates. The cells were incubated at 37 °C for 1 h, washed multiple times with PBS, and then supplemented with 2.5% NBS DMEM. The TCID50 was determined using the Karber’s method [14].

### 2.11. Cell Viability

HeLa cells were seeded into 96-well plates and treated with an inhibitor for 24 h after reaching full confluence. Subsequently, CCK-8 reagent (Vazyme Biotech, Nanjing, China) was added. Following a 4 h incubation at 37 °C, the absorbance was assessed at 450 nm using a microplate reader (Flash Spectrum Biotechnology, Shanghai, China).

### 2.12. Co-Immunoprecipitation (Co-IP)

Cells were collected and lysed in NP-40 buffer. Subsequently, 1 μg of a primary antibody was added to 400 μL of cell lysate and incubated overnight at 4 °C. Next, 20 μL of protein A+G agarose was added, and the mixture was gently shaken for 3 h at 4 °C before centrifugation. The supernatant was then carefully removed. The precipitate was washed five times with PBS, after which SDS-PAGE electrophoresis loading buffer was added and the sample was heated at 95 °C for 10 min to prepare for Western blot analysis.

### 2.13. Nuclear-Mass Separation Test

The cells in the six-well plate were collected, and 200 μL of pre-cooled CERI (Cytoplasmic Extraction Reagent I) (Thermo Fisher Scientific, Waltham, MA, USA) was added to the tube, oscillated for 15 s, and stood on ice for 10 min. Subsequently, 11 μL of pre-cooled CERII (Cytoplasmic Extraction Reagent II) (Thermo Fisher Scientific, Waltham, MA, USA) was added, oscillated for 5 s, and ice bathed for 1 min; re-oscillation was performed for 5 s, centrifugation was performed at 4 °C for 5 min, and rotation was performed at 16,000× *g*. The supernatant was transferred to the new tube (the supernatant was cytoplasmic protein and the precipitate was nuclear protein). After washing the precipitate with PBS, 100 μL pre-cooled NER (Nuclear Extraction Reagent) (Thermo Fisher Scientific, Waltham, MA, USA) was added to the precipitate, shaken for 15 s, put in an ice bath for 10 min, shaken again for 15 s, and centrifuged for 10 min at 16,000× *g*. An appropriate amount of 5× loading buffer was added to cytoplasmic protein and nuclear protein, respectively, and the samples were boiled at 95 °C for 5 min. The samples were stored at −80 °C for subsequent Western blot detection.

### 2.14. Ubiquitination Assay

The pcDNA3.1-3×flag-TRIM13 eukaryotic expression plasmid and pcDNA3.1-Myc-IκBα plasmid were co-transfected with pcDNA3.1-Myc-Ub, pcDNA3.1-Myc-K48, and pcDNA3.1-Myc-K63, respectively. The proteasome inhibitor MG132 (25 μM) was added at 4–6 h before cell collection, and the subsequent steps were the same as above in Section 2.13.

### 2.15. Statistical Analysis

The experimental results were obtained from three separate groups of experiments and analyzed using one-way ANOVA in GraphPad Prism Version 5.0. Significant differences in the data are denoted as * *p* < 0.05, ** *p* < 0.01, *** *p* < 0.001.

## 3. Results

### 3.1. TRIM13 Promotes the Proliferation of EMCV in HeLa Cells

Accumulating evidence identifies the tripartite motif (TRIM) protein family as pivotal regulators of the proliferation and dissemination of diverse viruses. Although TRIM13 has been characterized as a tumor suppressor in non-small-cell lung cancer (NSCLC) via NF-κB inhibition [13], its antiviral activity and underlying molecular mechanisms during encephalomyocarditis virus (EMCV) infection remain unknown.

**Figure 1:**
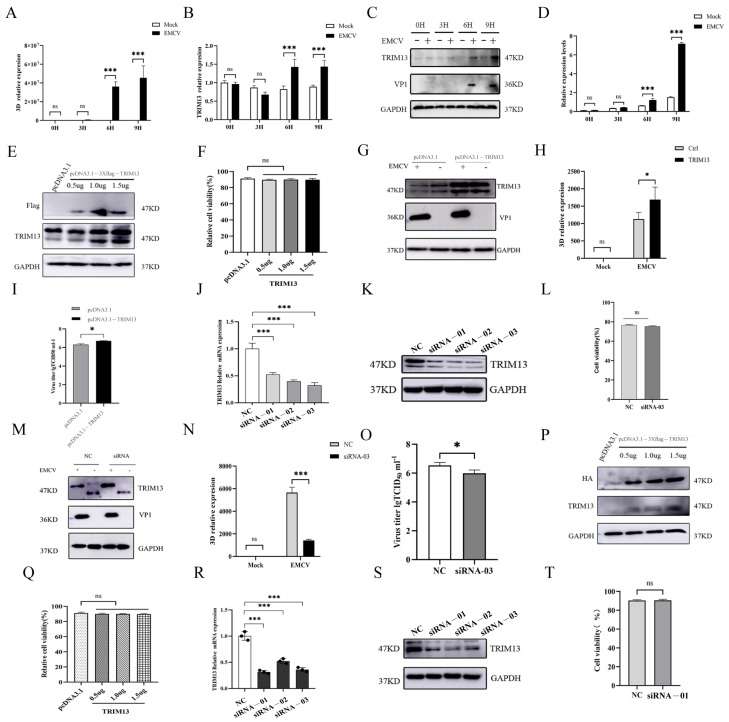
TRIM13 promotes the proliferation of EMCV. (**A**,**B**) HeLa cells infected with EMCV for 0 h, 3 h, 6 h or 9 h were collected; 3D (**A**) and TRIM13 (**B**) expression was detected by RT-qPCR. (**C**,**D**) HeLa cells infected with EMCV for 0 h, 3 h, 6 h or 9 h were collected, and TRIM13 expression was detected by Western blot and densitometric analysis. (**E**,**F**) HeLa cells were transfected with 0.5 μg, 1.0 μg, or 1.5 μg of pcDNA3.1-3×Flag-TRIM13 plasmid and harvested 48 h post-transfection (hpt). TRIM13 expression was assessed by Western blot analysis (**E**), and cell viability was quantified by CCK-8 assay (**F**). (**G**–**I**) HeLa cells were transfected with 1.5 μg of pcDNA3.1-3×Flag-TRIM3 plasmid and then infected with EMCV (MOI of 1), transferred to 37 °C for 1 h, and then transferred to 37 °C for 9 h; cells and supernatant were harvested. TRIM13 expression was analyzed by Western blot analysis (**G**), and EMCV proliferation was assessed by RT-qPCR (**H**) and viral titration (**I**). (**J**,**K**) HeLa cells were transfected with TRIM13-siRNA-01, TRIM13-siRNA-02 or TRIM13-siRNA-03 and harvested 48 hpt. TRIM13 expression was detected by RT-qPCR (**J**) and Western blot analysis (**K**). (**L**) HeLa cells were transfected with TRIM13-siRNA-03 and harvested 48 hpt, and cell viability was quantified by the CCK-8 assay. (**M**–**O**) HeLa cells were transfected with TRIM13-siRNA-03, bound with EMCV (MOI of 1), transferred to 37 °C for 1 h, and then transferred to 37 °C for 9 h; the cells and supernatant were collected. TRIM13 expression was detected by Western blot analysis (**M**), and EMCV proliferation was assessed by viral titration (**N**,**O**). (**P**,**Q**) BHK-21 cells were transfected with 0.5 μg, 1.0 μg, or 1.5 μg of pcDNA3.1-HA-TRIM1 plasmid and harvested 48 hpt. TRIM13 expression was assessed by Western blot analysis (**P**), and cell viability was quantified by the CCK-8 assay (**Q**). (**R**,**S**) BHK-21 cells were transfected with TRIM13-siRNA-01, TRIM13-siRNA-02 or TRIM13-siRNA-03 and harvested 48 hpt. TRIM13 expression was detected by RT-qPCR (**R**) and Western blot analysis (**S**). (**T**) BHK-21 cells were transfected with TRIM13-siRNA-01 and harvested 48 hpt, and cell viability was quantified by the CCK-8 assay. One-way analysis of variance (ANOVA) followed by Bonferroni multiple comparison of one independent experiment (*n* = 3). ns, not significant. * *p* < 0.05, *** *p* < 0.001.

To investigate the role of TRIM13 in EMCV replication, HeLa cells were infected with EMCV for 0, 3, 6, or 9 h. The viral capsid protein VP1 and the non-structural protein 3D have been validated as reliable markers for EMCV replication [15,16]. Notably, 3D represents the viral RNA-dependent RNA polymerase (RdRp), and its transcript level directly correlates with viral replication activity. Therefore, 3D and VP1 were employed as markers to monitor viral replication in all subsequent experiments. Reverse transcription quantitative PCR (RT-qPCR) analysis revealed that TRIM13 expression was upregulated during EMCV replication (Figure 1A,B). The results of the Western blot (Figure 1C) and densitometric analysis (Figure 1D) further confirmed that EMCV infection promoted TRIM13 expression. These results demonstrate that EMCV infection induces TRIM13 expression. To further investigate the effect of TRIM13 on EMCV replication, HeLa cells were transfected with increasing doses of pcDNA3.1-3×Flag-TRIM13 plasmid (0.5, 1.0, and 1.5 μg). The overexpression efficiency of TRIM13 was confirmed by immunoblot analysis (Figure 1E), and cell viability was monitored under the same transfection conditions (Figure 1F). The transfected cells were then infected with EMCV for 9 h. RT-qPCR and TCID_50_ assays demonstrated that TRIM13 overexpression significantly promoted EMCV replication (Figure 1H,I). As transfection with 1.5 μg of pcDNA3.1-TRIM13 yielded the highest TRIM13 expression level in HeLa cells, this dose was selected for subsequent experiments. Conversely, HeLa cells were transfected with three distinct TRIM13-specific siRNAs (Figure 1J,K), among which siRNA-03 most effectively silenced TRIM13 expression (Figure 1L). Subsequent infection with EMCV for 9 h revealed that TRIM13 knockdown significantly inhibited viral replication, as evidenced by RT-qPCR and TCID_50_ analyses (Figure 1M–O).

To determine whether the proviral effect of TRIM13 on EMCV replication is cell type-specific, parallel experiments were conducted in BHK-21 cells. Western blotting, CCK-8 assay, and RT-qPCR analysis confirmed successful TRIM13 overexpression in BHK-21 cells (Figure 1P–R). Similarly, transfection with three TRIM13-specific siRNAs identified siRNA-01 as the most effective for TRIM13 silencing in this cell line (Figure 1S,T).

Collectively, these results demonstrate that TRIM13 promotes EMCV replication.

### 3.2. TRIM13 Selectively Potentiates EMCV-Induced Pro-Inflammatory Cytokine Expression in HeLa Cells

We initially demonstrated that TRIM13 could promote EMCV proliferation by overexpressing and silencing TRIM13, followed by inoculation with EMCV. Consequently, we examined whether it concurrently modulates the host inflammatory response and whether this modulation exhibits host specificity at the cell or virus level.

Firstly, to investigate whether TRIM13 regulates the host inflammatory response during EMCV infection, HeLa cells were transfected with 1.5 µg of TRIM13-overexpression plasmid or siRNA-03 specifically targeting TRIM13, followed by EMCV infection for 9 h.

**Figure 2:**
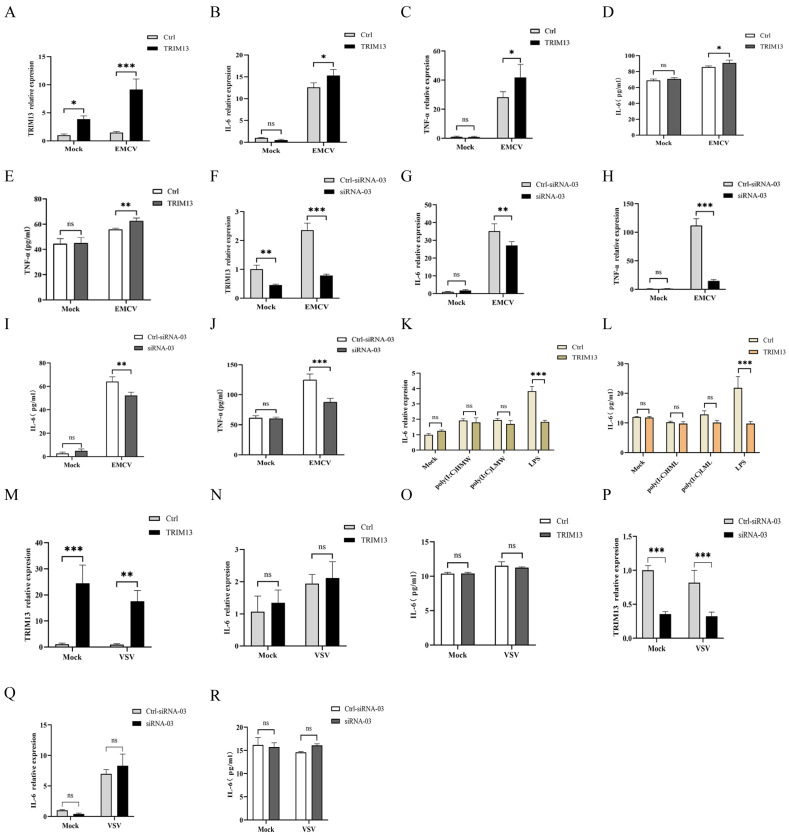
TRIM13 selectively potentiates EMCV-induced pro-inflammatory cytokine expression in HeLa cells. (**A**–**E**) HeLa cells were transfected with 1.5 μg of pcDNA3.1-3×Flag-TRIM13 plasmid and then infected with EMCV (MOI of 1), transferred to 37 °C for 1 h, and then transferred to 37 °C for 9 h; cells and supernatant were harvested. TRIM13 (**A**), IL-6 (**B**,**D**) and TNF-α (**C**,**E**) expression were assessed by RT-qPCR (**A**–**C**) analysis and ELISA analysis (**D**,**E**). (**F**–**J**) HeLa cells were transfected with siRNA-03 specifically targeting TRIM13 and then infected with EMCV (MOI of 1), transferred to 37 °C for 1 h, and then transferred to 37 °C for 9 h; cells and supernatant were harvested. TRIM13 (**F**), IL-6 (**G**,**I**) and TNF-α (**H**,**J**) expression were assessed by RT-qPCR (**F**–**H**) analysis and ELISA analysis (**I**,**J**). (**K**,**L**) HeLa cells were transfected with 1.5 μg of pcDNA3.1-3×Flag-TRIM3 plasmid and then transfected with poly(I:C)-HMW (4 µg/mL), poly(I:C)-LMW (4 µg/mL), or LPS (1 µg/mL) and harvested 24 hpi. IL-6 and TNF-α expression were analyzed by RT-qPCR analysis (**K**) and ELISA analysis (**L**). (**M**–**R**) TRIM13-overexpressing (**M**–**O**) and TRIM13-knockdown HeLa cells (**P**–**R**) were infected with VSV (MOI of 1) and harvested 9 hpi. IL-6 expression were analyzed by RT-qPCR analysis (**N**,**Q**) and ELISA analysis (**O**,**R**). TRIM13 expression levels in the indicated experimental groups, confirming efficient overexpression (**M**) or knockdown (**P**) prior to functional assays. One-way analysis of variance (ANOVA) followed by Bonferroni multiple comparison of one independent experiment (*n* = 3). ns, not significant. * *p* < 0.05, ** *p* < 0.01, *** *p* < 0.001.

To assess the inflammatory response induced by EMCV infection and the regulatory role of TRIM13, we prioritized the measurement of IL-6 and TNF-α, which represent the canonical NF-κB-dependent pro-inflammatory cytokines and are critical mediators of EMCV-induced pathogenesis [17]. These cytokines were selected based on their established roles as sensitive downstream markers of NF-κB activation and their rapid induction kinetics during the early phase of viral infection. RT-qPCR analysis revealed that TRIM13 overexpression significantly increased the transcript levels of IL-6 (Figure 2B) and TNF-α (Figure 2C) (overexpression efficiency shown in Figure 2A), whereas TRIM13 silencing significantly reduced these transcript levels (Figure 2G,H; knockdown efficiency shown in Figure 2F). ELISA quantification of secreted cytokines confirmed these transcriptional changes (Figure 2D,E,I,J).

To assess cell-type specificity, identical experiments were conducted in BHK-21 cells. TRIM13 overexpression did not significantly alter EMCV-induced IL-6 or TNF-α secretion (Figure S1A–E). Conversely, TRIM13 knockdown suppressed IL-6 expression but paradoxically enhanced TNF-α production (Figure S1F–J).

To elucidate the mechanism by which TRIM13 regulates inflammatory cytokine release, TRIM13-overexpressing or control HeLa cells were treated with poly(I:C)-HMW (4 µg/mL, TLR3 ligand), poly(I:C)-LMW (4 µg/mL, RIG-I ligand), or LPS (1 µg/mL, TLR4 ligand) for 9 h. The results of the RT-qPCR (Figure 1K) and ELISA (Figure 1L) analyses indicated that LPS treatment alone attenuated IL-6 expression, suggesting that TRIM13 does not globally potentiate pattern-recognition receptor signaling.

To determine whether TRIM13-mediated cytokine upregulation is virus-specific, TRIM13-overexpressing and TRIM13-knockdown HeLa cells were infected with vesicular stomatitis virus (VSV). RT-qPCR analysis showed that the mRNA levels of TRIM13 (Figure 2M,P) were significantly different compared with the control group. RT-qPCR analysis showed that the mRNA levels of IL-6 (Figure 2N,Q) did not differ significantly from those in the empty-vector control during VSV infection. ELISA analysis confirmed these findings (Figure 2O,R).

Collectively, these data demonstrate that TRIM13 modulates the host inflammatory response with cell-type and virus specificity during EMCV infection.

### 3.3. TRIM13 Promotes EMCV Proliferation and Regulates NF-κB Signaling

Previous studies have suggested that TRIM13 plays a positive regulatory role in the EMCV-induced inflammatory response. To elucidate the underlying mechanisms of TRIM13-mediated modulation, we investigated its involvement in antiviral signaling pathways. Accumulating evidence indicates that TRIM proteins can modulate antiviral immunity by regulating the NF-κB signaling pathway, either positively or negatively [11,12,18]. Based on these findings, we hypothesized that TRIM13 may regulate EMCV-triggered inflammation via the NF-κB pathway. To test this hypothesis, we examined whether TRIM13 influences key events such as p65 nuclear translocation or IκBα degradation, thereby clarifying its mechanistic role in EMCV infection.

#### 3.3.1. TRIM13 Regulates MDA5 and MAVS Expression

**Figure 3:**
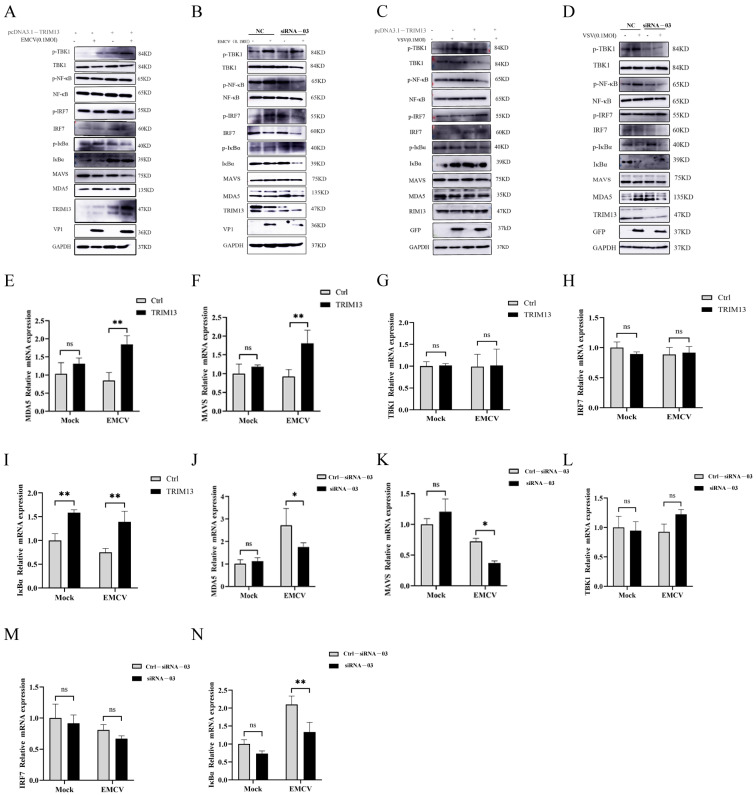
TRIM13 promotes EMCV proliferation by regulating the NF-κB pathway. (**A**,**B**) HeLa cells were transfected with pcDNA3.1-3×Flag-TRIM13 plasmid or TRIM13 siRNA-03 plasmid and then infected with EMCV (MOI of 1), transferred to 37 °C for 1 h, and then transferred to 37 °C for 9 h; cell pellets were harvested to detect the level of protein changes. (**C**,**D**) HeLa cells were transfected with pcDNA3.1-3×Flag-TRIM13 plasmid or TRIM13 siRNA-03 plasmid and then infected with VSV (MOI of 1), transferred to 37 °C for 1 h, and then transferred to 37 °C for 9 h; cell pellets were harvested to detect the level of protein changes. (**E**–**I**) HeLa cells were transfected with pcDNA3.1-3×Flag-TRIM13 plasmid and then infected with EMCV (MOI of 1), transferred to 37 °C for 1 h, and then transferred to 37 °C for 9 h; cell pellets were harvested. MDA5 (**E**), MAVS (**F**), TBK1 (**G**), IRF7 (**H**) and IκBα (**I**) expressions were assessed by RT-qPCR. (**J**–**N**) HeLa cells were transfected with TRIM13 siRNA-03 plasmid and then infected with EMCV (MOI of 1), transferred to 37 °C for 1 h, and then transferred to 37 °C for 9 h; cell pellets were harvested. MDA5 (**J**), MAVS (**K**), TBK1 (**L**), IRF7 (**M**) and IκBα (**N**) expressions were assessed by RT-qPCR. One-way analysis of variance (ANOVA) followed by Bonferroni multiple comparison of one independent experiment (*n* = 3). ns, not significant. * *p* < 0.05, ** *p* < 0.01.

HeLa cells were transfected with TRIM13-overexpression plasmid or TRIM13-specific siRNA-03, and subsequently infected with EMCV for 9 h. Cell pellets were collected to quantify the total and phosphorylated protein levels of MDA5, MAVS, TBK1, IRF7, NF-κB p65, and IκBα. Western blot analysis (Figure 3A,B) and RT–qPCR (Figure 3E–H) revealed that, in the absence of EMCV challenge, neither TRIM13 overexpression nor TRIM13 knockdown altered endogenous MDA5 (Figure 3E,J) and MAVS (Figure 3F,K) expression. However, upon EMCV infection, TRIM13 overexpression significantly upregulated MDA5 (Figure 3E) and MAVS (Figure 3F) expression, whereas TRIM13 knockdown markedly reduced their expression (Figure 3J,K). Notably, EMCV infection induced TBK1 (Figure 3G,L) and IRF7 (Figure 3H,M) in HeLa cells regardless of TRIM13 overexpression or knockdown, indicating that TRIM13 does not regulate the activation of these signaling molecules. These findings are consistent with our previous observations following VSV infection (Figure 3C,D).

#### 3.3.2. TRIM13 Modulates the NF-κB Signaling Pathway via IκBα Regulation

The NF-κB signaling axis is stringently governed by IκBα and functions as a central regulator of both innate and adaptive immunity [19]. To determine whether TRIM13 modulates NF-κB activation, HeLa cells were transfected with TRIM13-overexpression plasmid or TRIM13-specific siRNA-03, and subsequently infected with EMCV for 9 h. The results demonstrated that TRIM13 overexpression significantly upregulated IκBα expression and concomitantly downregulated its phosphorylation level (Figure 3I). Conversely, TRIM13 knockdown resulted in downregulated IκBα expression (Figure 3N). These findings confirm that TRIM13 participates in EMCV-induced NF-κB-related signaling pathway activation by regulating MDA5, MAVS, and IκBα, but does not affect TBK1 or IRF7 protein expression or phosphorylation levels. Collectively, these results indicate that TRIM13 does not modulate the TBK1-IRF7 signaling pathway.

#### 3.3.3. TRIM13-Mediated Regulation Is Independent of TRAF and IKK Complexes

Tumor necrosis factor receptor-associated factor 3 (TRAF3) acts as a positive regulator of type I interferon production, while inhibiting the MAPK cascade and the non-canonical NF-κB axis [20,21]. Another member of the TRAF family, TRAF6, serves as an important adaptor molecule for canonical NF-κB activation and has also been reported to negatively regulate TNF-α-mediated NF-κB signaling [22]. Additionally, IKKα and IKKβ kinases can form IKK complexes that play a critical role in IκB phosphorylation and NF-κB activation [23]. Based on these observations, we further investigated whether TRAF and IKK complexes are involved in TRIM13-mediated regulation of EMCV infection. RT–qPCR analysis revealed that the transcription levels of TRAF3, TRAF6, IKKα, and IKKβ remained unaffected by TRIM13 overexpression or knockdown following EMCV infection (Figure S2A–D,G–J), indicating that TRIM13 regulates virus-induced inflammatory response is not related to TRAFs and IKK complexes.

#### 3.3.4. TRIM13-Mediated Regulation Is Independent of the JAK-STAT Pathway

Interferons (IFNs) bind to IFN receptors on the surface of adjacent cells, induce the expression of interferon-stimulated genes (ISGs) and transcriptional regulators, and activate the JAK-STAT pathway, thereby exerting multiple functions including antiviral activity, immune regulation, and activation of natural killer cells [24,25]. Our previous study demonstrated that the C protein of Peste des petits ruminants virus (PPRV) suppresses interferon (IFN) production by inhibiting STAT1 phosphorylation [26]. Based on this observation, we hypothesized that TRIM13 might modulate viral replication via the JAK-STAT pathway. To evaluate this possibility, HeLa cells were transfected with TR-IM13 overexpression or knockdown constructs, and subsequently infected with EMCV. RT–qPCR analysis revealed no significant changes in JAK1 (Figure S2E,F) or S-TAT1 (Figure S2K,L) mRNA levels compared to controls under either mock or EMCV-infected conditions.

Collectively, these findings demonstrate that TRIM13 enhances EMCV proliferation in parallel with NF-κB activation.

### 3.4. TRIM13 Targets IκBα to Regulate the NF-κB Pathway

To identify the potential targets of TRIM13 in the NF-κB signaling pathway, HeLa cells were co-transfected with the internal reference plasmid pRL-TK and the reporter gene plasmid pGL3-NF-κB. Cells were co-transfected with empty pcDNA3.1 (vector) or pcDNA3.1-TRIM13 together with the indicated expression plasmids encoding Flag-MDA5, Flag-IRF7, Flag-MAVS, Flag-TBK1, Flag-IKKα, Flag-IKKβ, Flag-TRAF6, Flag-TRAF3, or Myc-IκBα.

**Figure 4:**
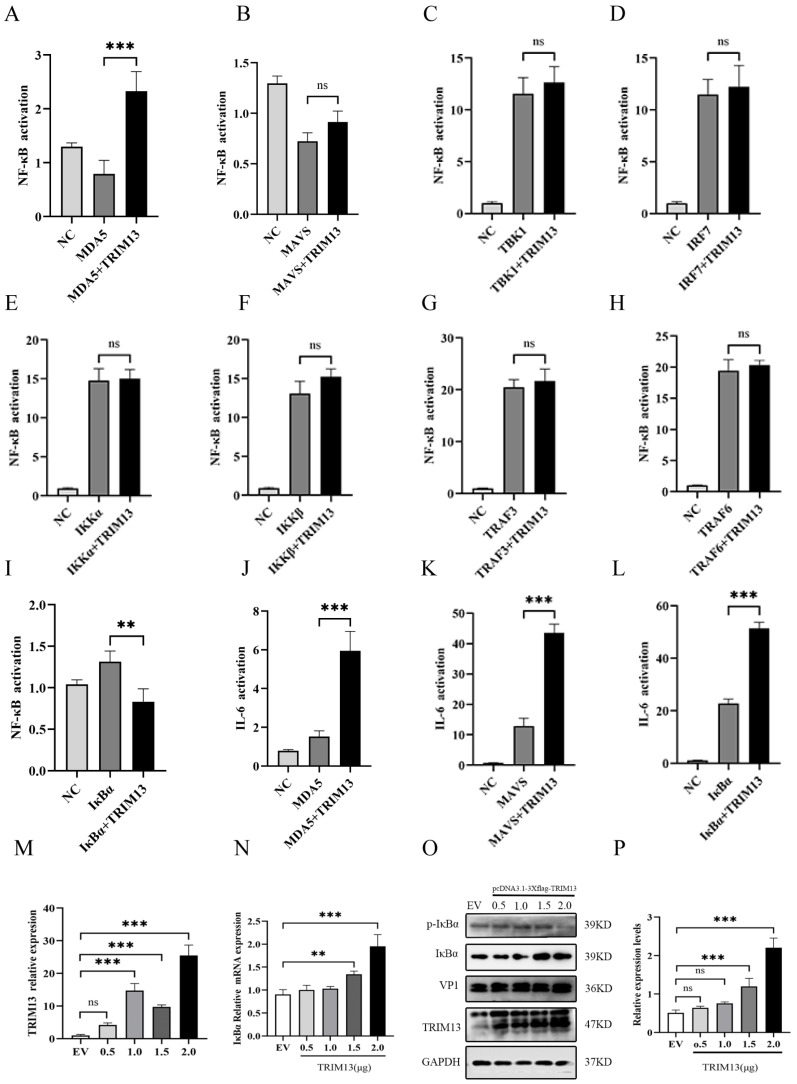
TRIM13 promotes EMCV proliferation by regulating the NF-κB pathway. (**A**–**I**) HeLa cells were co-transfected with empty pcDNA3.1 (vector) or pcDNA3.1-TRIM13 together with the indicated expression plasmids encoding Flag-MDA5, Flag-IRF7, Flag-MAVS, Flag-TBK1, Flag-IKKα, Flag-IKKβ, Flag-TRAF6, Flag-TRAF3 or Myc-IκBα for 48 h; cell pellets were harvested. Luciferase reporter assays were employed to evaluate the regulatory roles of MDA5 (**A**), MAVS (**B**), TBK1 (**C**), IRF7 (**D**), IKKα (**E**), IKKβ (**F**), TRAF3 (**G**), TRAF6 (**H**) or IκBα (**I**) on NF-κB promoters. (**J**–**L**) HeLa cells were co-transfected with empty pcDNA3.1 (vector) or pcDNA3.1-TRIM13 together with the indicated expression plasmids encoding Flag-MDA5, Flag-MAVS or Myc-IκBα for 48 h; cell pellets were harvested. Luciferase reporter assays were employed to evaluate the regulatory effects of MDA5 (**J**), MAVS (**K**) or IκBα (**L**) on IL-6 promoters. (**M**,**N**) HeLa cells were transfected with 0.5 μg, 1.0 μg, or 1.5 μg of pcDNA3.1-3×Flag-TRIM13 plasmid and harvested 48 hpt. TRIM13 (**M**) and IκBα (**N**) expression were assessed by RT-qPCR analysis. (**O**,**P**) HeLa cells were transfected with 0.5 μg, 1.0 μg, or 1.5 μg of pcDNA3.1-3×Flag-TRIM13 plasmid and harvested 48 hpt. TRIM13, IκBα, p- IκBα and VP1 expression were assessed by Western blot (**O**) and densitometric analysis (**P**). One-way analysis of variance (ANOVA) followed by Bonferroni multiple comparison of one independent experiment (*n* = 3). ns, not significant. ** *p* < 0.01, *** *p* < 0.001.

Dual-luciferase assays revealed that MDA5 overexpression alone activated the RIG-I-like receptor (RLR) axis and elevated NF-κB reporter activity 2.0-fold relative to the vector control; co-expression of TRIM13 further augmented this induction to approximately twice the MDA5-only level (Figure 4A). In contrast, individual overexpression of MAVS (Figure 4B), TBK1 (Figure 4C), IRF7 (Figure 4D), IKKα (Figure 4E), IKKβ (Figure 4F), TRAF3 (Figure 4G) or TRAF6 (Figure 4H) each increased NF-κB promoter activity; however, TRIM13 co-expression failed to enhance these responses further. Notably, IκB-α overexpression produced a 20-fold increase in NF-κB reporter activity compared with the vector control, whereas TRIM13 co-expression markedly inhibited this activation (Figure 4I).

To further delineate the target of TRIM13 within the inflammatory axis, the impact of MDA5, MAVS, and IκBα on IL-6 promoter activity was assessed. pcDNA3.1 and pcDNA3.1-TRIM13 were co-transfected with pRK-Flag-MDA5, pRK-Flag-MAVS, or pcDNA3.1-Myc-IκBα, respectively, and analyzed using the dual-luciferase reporter system. Overexpression of MDA5 (Figure 4J), MAVS (Figure 4K) and IκBα (Figure 4L) in the positive control groups increased IL-6 promoter activity, and co-expression of TR-IM13 significantly enhanced these effects compared with the respective positive controls.

Phosphorylation of IκBα serves as a hallmark of NF-κB pathway activation. To further investigate whether IκBα constitutes a target of TRIM13-mediated inflammatory regulation, TRIM13 was overexpressed in HeLa cells, and IκBα expression was subsequently analyzed. RT-qPCR analysis demonstrated that IκBα mRNA levels (Figure 4N) increased concomitantly with TRIM13 expression (Figure 4M). Western blot analysis (Figure 4O) and densitometric quantification (Figure 4P) further confirmed that TRIM13 promoted IκBα protein expression.

These results indicate that TRIM13 positively regulates EMCV replication and is accompanied by increased IκBα expression, suggesting a potential regulatory association between them.

### 3.5. TRIM13 Promotes Viral Replication by Catalyzing IκBα Ubiquitination

**Figure 5:**
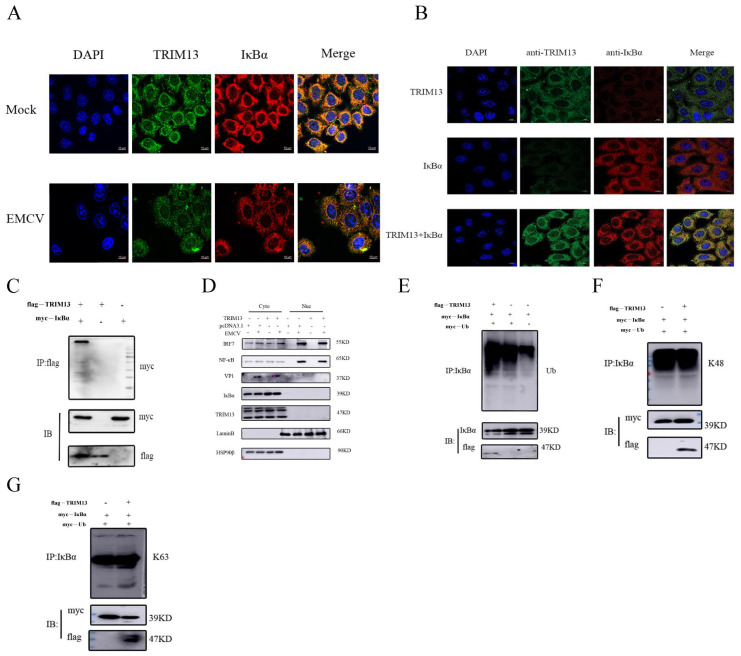
TRIM13 promotes IκBα ubiquitination. (**A**) Co-localization of TRIM13 and IκBα proteins in HeLa cells and then infected with EMCV for 9 h. (**B**) Co-localization of TRIM13 and IκBα proteins in HeLa cells expressing TRIM13 and IκBα for 48 h. Nuclei were stained using DAPI, respectively. Data are representative of at least three independent experiments. (**C**) Co-IP and IB analysis of the interaction between TRIM13 and IκBα. (**D**) HeLa cells were transfected with pcDNA3.1-3×Flag-TRIM13 plasmid and subsequently infected with EMCV (MOI of 1). Nuclear–cytoplasmic fractionation was performed, and the subcellular distribution of VP1, TRIM13, IκBα, NF-κB, IRF7, Lamin B1 and GAPDH for cytoplasmic was analyzed by Western blot. Loading controls: Lamin B1 (nuclear fraction) and GAPDH (cytoplasmic fraction). (**E**) Immunoprecipitation analysis to investigate the regulatory effect of TRIM13 on the ubiquitination status of IκBα. (**F**) Immunoprecipitation assay to assess the influence of TRIM13 on K48-linked polyubiquitination of IκBα. (**G**) Immunoprecipitation experiment to evaluate the impact of TRIM13 on K63-linked polyubiquitination of IκBα.

To investigate whether TRIM13 directly targets IκBα, we first examined their subcellular localization in HeLa cells. Confocal microscopy revealed that TRIM13 and IκBα co-localized in the cytoplasm under normal conditions; however, this co-localization was markedly reduced following EMCV infection (Figure 5A). To further validate this interaction, we performed co-transfection experiments and observed pronounced spatial overlap between the two proteins exclusively when both constructs were present, with no detectable signal overlap in single transfections (Figure 5B), supporting a specific TRIM13–IκBα association.

We next confirmed the physical interaction between TRIM13 and IκBα using co-immunoprecipitation (co-IP) assays in HEK293T cells. Cells were transiently transfected with Flag-tagged TRIM13 and Myc-tagged IκBα expression vectors, and lysates were subjected to immunoprecipitation with an anti-Myc antibody. The results demonstrated that Flag-TRIM13 was efficiently co-precipitated with Myc-IκBα, confirming their interaction (Figure 5C).

Given that TRIM13 functions as an E3 ubiquitin ligase and that the NF-κB pathway is tightly regulated by the ubiquitin-proteasome system [27,28], we hypothesized that TRIM13 might modulate IκBα stability through ubiquitination. Upon pathway activation, IκBα undergoes phosphorylation, followed by ubiquitination and proteasomal degradation, thereby releasing NF-κB for nuclear translocation and transcription of target genes [29]. To test this hypothesis, we examined whether TRIM13 affects IκBα ubiquitination. Overexpression of TRIM13 enhanced IκBα ubiquitination levels (Figure 5E).

Ubiquitination occurs through diverse linkages, including monoubiquitination and polyubiquitin chains formed via lysine residues (K6, K11, K27, K29, K33, K48, and K63) [14]. Notably, K48-linked chains typically mediate proteasomal degradation, whereas K63-linked chains primarily regulate signal transduction [30,31]. To determine the specific ubiquitin linkage type involved in TRIM13-mediated IκBα modification, we performed additional analyses. Surprisingly, our results indicated that TRIM13-induced IκBα ubiquitination was not mediated by K48- or K63-linked polyubiquitination, as determined using antibodies specific for K48- and K63-linked polyubiquitin chains (Figure 5F,G), suggesting the involvement of alternative ubiquitin linkage types.

Finally, we examined the subcellular distribution of key pathway components during EMCV infection. Nuclear–cytoplasmic fractionation analysis revealed that EMCV infection induced activation of the inflammatory pathway, as evidenced by nuclear translocation of NF-κB and IRF7, whereas TRIM13 and IκBα remained predominantly cytoplasmic (Figure 5D). This observation was consistent with our immunofluorescence assay (IFA) results (Figure 5A). The nuclear accumulation of NF-κB correlated with enhanced secretion of pro-inflammatory cytokines, consistent with our findings in Section 3.2.

In summary, these data demonstrate that TRIM13 functions as a critical positive regulator of EMCV-triggered inflammation. Mechanistically, TRIM13 interacts with IκBα and promotes its ubiquitination, thereby facilitating disruption of the IκBα–NF-κB complex and enabling NF-κB nuclear translocation. This selective activation of the NF-κB signaling axis ultimately enhances the production of IL-6 and TNF-α. Although TRIM13-mediated ubiquitination of IκBα is essential for NF-κB activation, the precise ubiquitin linkage type remains to be defined.

## 4. Discussion

Encephalomyocarditis virus (EMCV), a non-enveloped RNA virus belonging to the family *Picornaviridae* and genus *Cardiovirus* [1], primarily infects mammals, including rodents and primates, via the faecal–oral route, causing encephalomyocarditis (EMC) [2,3].

Tripartite motif (TRIM) proteins have emerged as critical regulators of antiviral immunity. These proteins typically function as E3 ubiquitin ligases, mediating the ubiquitination and subsequent proteasomal degradation of viral proteins or modulation of host signaling pathways [32,33,34,35,36] For instance, TRIM69 restricts dengue virus (DENV) replication by interacting with the viral non-structural protein NS3 and promoting its ubiquitin–proteasome degradation [32]. Similarly, TRIM22 exhibits antiviral activity against influenza A virus (IAV) by targeting the viral nucleoprotein (NP) for polyubiquitination and proteasomal degradation [33]. TRIM52 also demonstrates E3 ligase-dependent antiviral function by degrading Japanese encephalitis virus (JEV) NS2A through the proteasome pathway [34]. In addition to targeting viral proteins directly, TRIM proteins can modulate host immune signaling. Notably, TRIM5α restricts human immunodeficiency virus (HIV) replication by binding to the viral protease NS2B/3 and promoting its K48-linked ubiquitination and proteasomal degradation [35]. Furthermore, TRIM22 has been implicated in regulating the nuclear factor-kappa B (NF-κB) signaling pathway—a critical mediator of innate immunity and inflammation—by modulating the ubiquitination status of key regulatory proteins, including IκBα and IKKγ [36]. The NF-κB pathway comprises canonical (classical) and non-canonical (non-classical) branches, both of which are tightly regulated by post-translational modifications of IκBα [36].

TRIM13, an ER-associated E3 ubiquitin ligase, has been shown to suppress the inflammatory response induced by pathogenic DNA [11]. This finding provides a theoretical foundation for understanding the regulatory role of TRIM13 in EMCV-induced inflammatory responses.

In this study, we demonstrate that TRIM13 negatively regulates EMCV infection through modulation of the NF-κB signaling pathway. Overexpression of TRIM13 enhanced IκBα ubiquitination and degradation, thereby facilitating NF-κB nuclear translocation. These findings demonstrate that TRIM13 acts as an activator of NF-κB signaling in EMCV-infected cells.

Concurrently, we observed that TRIM13 modulates the expression of MDA5 and MAVS following viral infection. MDA5 serves as a critical cytosolic pattern recognition receptor that detects viral RNA and transmits signals through the adaptor protein MAVS. Upon activation, MAVS recruits downstream signaling components, including TBK1, which undergoes phosphorylation and subsequent activation. Previous studies have established that TBK1 contributes to NF-κB activation primarily by acting upstream of NIK and IKK complexes [37]. Specifically, TBK1 has been shown to induce IκB degradation and enhance NF-κB activity through IKKβ-mediated phosphorylation. However, our results indicate that although TBK1 is activated during EMCV infection, it does not participate in the TRIM13-mediated regulatory process, suggesting the existence of alternative signaling mechanisms.

Interestingly, luciferase reporter assays revealed that TRIM13 promotes NF-κB activation mediated by MDA5 while inhibiting NF-κB activation mediated by IκBα. Notably, individual overexpression of TBK1, TRAF3, IRF7, IKKα, IKKβ, or TRAF6 did not significantly affect NF-κB promoter activity in our experimental system. Furthermore, TRIM13 enhanced IL-6 activation mediated by MDA5, MAVS, and IκBα. Collectively, these results lead us to hypothesize that MDA5, MAVS, and IκBα may serve as potential target proteins for TRIM13-mediated regulation during viral infection.

In the immune response, post-translational modifications, particularly ubiquitination, serve as critical regulatory mechanisms that orchestrate various stages of innate immunity. Our study identified TRIM13 as an E3 ubiquitin ligase that interacts with IκBα, promoting its ubiquitin–proteasome-dependent degradation. Co-localization and co-immunoprecipitation experiments confirmed the physical interaction between TRIM13 and IκBα. Notably, TRIM13-mediated ubiquitination of IκBα facilitated NF-κB nuclear translocation, subsequently enhancing the expression of pro-inflammatory cytokines IL-6 and TNF-α, which is consistent with our initial observations. Ubiquitination modifications occur at multiple lysine residues, including K6, K11, K27, K29, K33, K48, and K63 [38]. K48-linked polyubiquitination typically targets proteins for proteasomal degradation, whereas K63-linked ubiquitination primarily regulates signal transduction, endocytosis, inflammatory responses, and DNA repair [39]. Our findings demonstrated that TRIM13 promotes IκBα ubiquitination; however, further analysis revealed that this modification was independent of K48 and K63 linkages. The specific ubiquitination sites mediated by TRIM13 remain to be elucidated and warrant future investigation.

While the present study establishes the molecular mechanism of TRIM13 in EMCV infection using well-characterized cell culture models (HeLa and BHK-21 cells), we acknowledge that these findings require validation in primary cells and in vivo models to fully assess their physiological relevance. Transformed cell lines, despite their experimental advantages in mechanistic studies, may not fully recapitulate the complex tissue microenvironment and immune responses observed in natural EMCV infections. Future studies will investigate the role of TRIM13 in primary porcine cells and EMCV-infected animal models to confirm the pathophysiological significance of our findings.

In summary, this study reveals a novel regulatory mechanism whereby TRIM13 functions as an E3 ubiquitin ligase to promote IκBα ubiquitination and degradation, thereby activating NF-κB signaling. Our results suggest that TRIM13 is associated with NF-κB activation in HeLa cells, which may be linked to enhanced EMCV infection. The-se findings position TRIM13 as a potential therapeutic target; inhibitors targeting its E3 ligase activity or the TRIM13-IκBα interaction may represent promising strategies for treating inflammatory diseases. In addition, TRIM13 was able to significantly induce IL-6 reporter activity, implying that regulatory elements within the IL-6 promoter are targeted by TRIM13-mediated signaling pathways. However, the functional domains of TRIM13 remain poorly characterized and require further investigation. Additionally, the broader role of TRIM13 in inflammatory responses needs to be comprehensively evaluated to fully realize its therapeutic potential.

## Figures and Tables

**Table 1:** The sequences of the siRNAs, primers, and probe.

Name	Sequence (5′–3′)
TRIM13-siRNA-01 (human, Gene ID: 10206) ^1^	GCAAGCCCTTTAATGAAGA
TRIM13-siRNA-02 (human, Gene ID: 10206)	GTCCTACCATGTTCCTAGA
TRIM13-siRNA-03 (human, Gene ID: 10206)	GTGGAAAAGTATAACAAGA
TRIM13-siRNA-01 (hamster, Gene ID: 101824537) ^2^	CGATGTGGATAAACTTTCT
TRIM13-siRNA-02 (hamster, Gene ID: 101824537)	CCCTCTAACTTGCCCACAA
TRIM13-siRNA-03 (hamster, Gene ID: 101824537)	TGGAGTCAATAGTCTACAA

^1^ TRIM13-siRNA-01/02/03 (human, Gene ID 10206) were used for HeLa cells. ^2^ TRIM13-siRNA-01/02/03 (hamster, Gene ID 101824537) were used for BHK-21 cells.

**Table 2:** The fluorescence quantitative PCR primers were designed as follows.

Primer Name	Primer Sequence (5′ to 3′)
Human-GAPDH-qR	AGGGGCCATCCACAGTCTTC
Human-GAPDH-qF	AGAAGGCTGGGGCTCATTTG
Human-TRIM13-qR	GAATGTGCCAGTGTCTTGAGGC
Human-TRIM13-qF	CCTCCCTCTAATTTGCCTGCAAG
Mouse-GAPDH-qR	CTCGCTCCTGGAAGATGGTG
Mouse-GAPDH-qF	GGTGAAGGTCGGTGTGAACG
Mouse-TRIM13-qR	CACAGGCATGTTCATTAGCAAGATTC
Mouse-TRIM13-qF	GAGGAGACCCAGCAGAACTACC
EMCV-3D-qR	CATCTGTACTCCACACTCTCGAATG
EMCV-3D-qF	GTCATACTATCGTCCAGGGACTCTAT
VSV-G-qR	CGAAGCAGCGTCTTGAATGTGAG
VSV-G-qF	GGACCCAATGGAGTTCTGAGGAC
Human-GAPDH-qR	AGGGGCCATCCACAGTCTTC
Human-GAPDH-qF	AGAAGGCTGGGGCTCATTTG
Homo-IL-6-qF	CTCAATATTAGAGTCTCAACCCCCA
Homo-IL-6-qR	GAGAAGGCAACTGGACCGAA
Homo-TNFα-qF	AGAACTCACTGGGGCCTACA
Homo-TNFα-qR	GCTCCGTGTCTCAAGGAAGT
Mouse-GAPDH-qR	CTCGCTCCTGGAAGATGGTG
Mouse-GAPDH-qF	GGTGAAGGTCGGTGTGAACG
Mouse-TNF-qR	CCCGTCGCAAAGTTTCTGTAAGTG
Mouse-TNF-qF	CACCTCACCGCCAAGTCTCAC
Mouse-IL-6-qR	GTTGGGAGTAGTGTCCTCTGTGAAG
Mouse-IL-6-qF	TTGGGACTGCTGCTGGTGATG

## Data Availability

The original contributions presented in the study are included in the article. Further inquiries can be directed to the corresponding author.

## Supplementary Materials

The following supporting information can be downloaded at: https://www.mdpi.com/article/10.3390/v18040466/s1, Figure S1. TRIM13 selectively potentiates EMCV-induced pro-inflammatory cytokine expression in BHK-21 cells. (A–E) BHK-21 cells were transfected with 1.5 μg of pcDNA3.1-3×Flag-TRIM1 plasmid and then infected with EMCV (MOI of 1), transferred to 37 °C for 1 h, and then transferred to 37 °C for 9 h, cells and supernatant were harvested. TRIM13 (A), IL-6 (B,D) and TNF-α (C,E) expression were assessed by RT-qPCR (A–C) analysis and ELISA analysis (D,E). (F–J) BHK-21 cells were transfected with siRNA-03 specifically targeting TRIM13 and then infected with EMCV (MOI of 1), transferred to 37 °C for 1 h, and then transferred to 37 °C for 9 h, cells and supernatant were harvested. TRIM13 (F), IL-6 (G,I) and TNF-α (H,J) expression were assessed by RT-qPCR (F-H) analysis and ELISA analysis (I,J). one-way analysis of variance (ANOVA) followed by Bonferroni multiple comparison of one independent experiment (*n* = 3). One representative experiment of two is shown. ns, not significant. * *p* < 0.05, ** *p* < 0.01, *** *p* < 0.001. Figure S2. TRIM13 promotes EMCV proliferation by regulating the NF-κB pathway. (A–F) HeLa cells were transfected with pcDNA3.1-3×Flag-TRIM1 plasmid or TRIM13 siRNA-03 plasmid and then infected with EMCV (MOI of 1), transferred to 37 °C for 1 h, and then transferred to 37 °C for 9 h, cell precipitation was harvested. TRAF3 (A), TRAF6 (B), IKKα (C), IKKβ (D), JAK11(E), STAT1 (F) expressions were assessed by RT-qPCR. (G to L) HeLa cells were transfected with TRIM13 siRNA-03 plasmid and then infected with EMCV (MOI of 1), transferred to 37 °C for 1 h, and then transferred to 37 °C for 9 h, cell precipitation was harvested. TRAF3 (G), TRAF6 (H), IKKα (I), IKKβ (J), JAK11(K), STAT1 (L) expressions were assessed by RT-qPCR. one-way analysis of variance (ANOVA) followed by Bonferroni multiple comparison of one independent experiment (*n* = 3). One representative experiment of two is shown. ns, not significant. * *p* < 0.05, ** *p* < 0.01, *** *p* < 0.001.
