# Supplementary material for: TRIM13 Positively Regulates the NF-κB Signaling Pathway Induced by Encephalomyocarditis Virus
Source: Viruses. 2026 Apr 14;18(4):466. doi: 10.3390/v18040466 (PMC13120059; doi:10.3390/v18040466)
Supplement: Supplementary file 1 [file viruses-18-00466-s001.zip › viruses-4214597-supplementary.pdf]

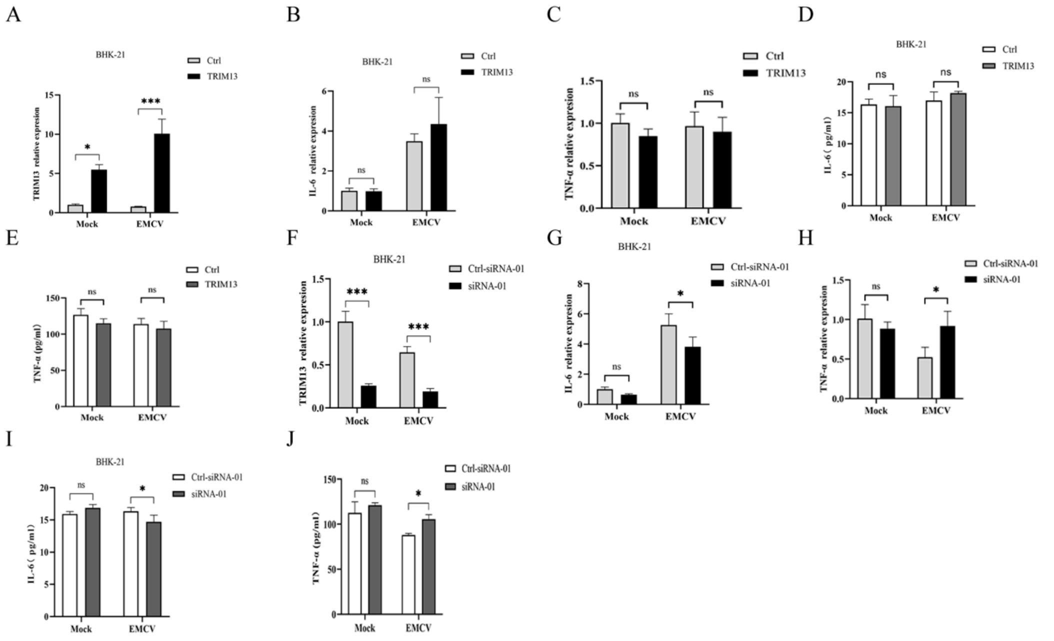

Figure S1. TRIM13 selectively potentiates EMCV-induced pro-inflammatory cytokine expression in BHK-21 cells. (A-E) BHK-21 cells were transfected with 1.5 μg of pcDNA3.1-3×Flag-TRIM1 plasmid and then infected with EMCV (MOI of 1), transferred to 37 ℃ for 1 h, and then transferred to 37 ℃ for 9 h, cells and supernatant were harvested. TRIM13 (A), IL-6 (B,D) and TNF-α (C,E) expression were assessed by RT-qPCR (A to C) analysis and ELISA analysis (D,E). (F to J) BHK-21 cells were transfected with siRNA-03 specifically targeting TRIM13 and then infected with EMCV (MOI of 1), transferred to 37 ℃ for 1 h, and then transferred to 37 ℃ for 9 h, cells and supernatant were harvested. TRIM13 (F), IL-6 (G,I) and TNF-α (H,J) expression were assessed by RT-qPCR (F-H) analysis and ELISA analysis (I,J). one-way analysis of variance (ANOVA) followed by Bonferroni multiple comparison of one independent experiment (n =1). One representative experiment of two is shown. ns, not significant. *$p < 0.05$, **$p < 0.01$, ***$p < 0.001$.

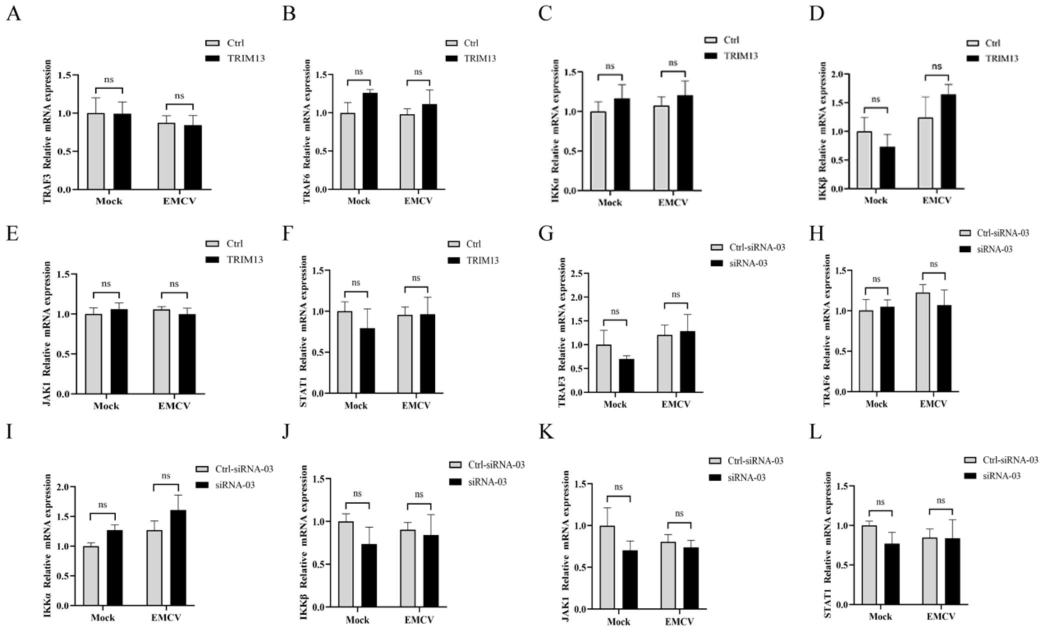

Figure S2. TRIM13 promotes EMCV proliferation by regulating the NF-κB pathway. (A to F) HeLa cells were transfected with pcDNA3.1-3×Flag-TRIM1 plasmid or TRIM13 siRNA-03 plasmid and then infected with EMCV (MOI of 1), transferred to 37 ℃ for 1 h, and then transferred to 37 ℃ for 9 h, cell precipitation was harvested. TRAF3 (A), TRAF6 (B), IKK*α* (C), IKKβ (D), JAK11(E), STAT1 (F) expressions were assessed by RT-qPCR. (G to L) HeLa cells were transfected with TRIM13 siRNA-03 plasmid and then infected with EMCV (MOI of 1), transferred to 37 ℃ for 1 h, and then transferred to 37 ℃ for 9 h, cell precipitation was harvested. TRAF3 (G), TRAF6 (H), IKK*α* (I), IKKβ (J), JAK11(K), STAT1 (L) expressions were assessed by RT-qPCR. one-way analysis of variance (ANOVA) followed by Bonferroni multiple comparison of one independent experiment (n =1). One representative experiment of two is shown. ns, not significant. *$p < 0.05$, **$p < 0.01$, ***$p < 0.001$.
